# The effect of external bleeding control training courses on lay first-person responders'knowledge, skills, and attitudes in low- and middle-income countries: a systematic review

**DOI:** 10.1007/s00068-025-02917-4

**Published:** 2025-07-14

**Authors:** Husham Abdelrahman, Ahammed Mekkodathil, Ayman El-Menyar, Rafael Consunji, Sandro Rizoli, Hassan Al-Thani

**Affiliations:** 1 Department of Surgery, Trauma Surgery, Hamad Medical Corporation, Doha, Qatar; 2 Department of Surgery, Clinical Research, Trauma & Vascular Surgery, Hamad Medical Corporation, Doha, Qatar; 3 Department of Clinical Medicine, Weill Cornell Medical College, Doha, Qatar; 4 Department of Surgery, Trauma Surgery, Injury Prevention, Hamad Medical Corporation, Doha, Qatar

**Keywords:** Hemorrhage control, Trauma, Injury, Training, Low- and middle-income country, Layperson, Systematic review

## Abstract

**Background:**

Exsanguinating hemorrhage is the most common cause of preventable trauma death at the injury scene, and it is often due to the inability to control bleeding immediately. Training layperson first responders (LFR) in trauma care, particularly hemorrhage control, has been recommended to address this trauma care gap. We conducted a systematic review (SR) to analyze the effect of hemorrhage control training courses for LFRs on knowledge, skill, and attitude to intervene in trauma patients.

**Methods:**

PubMed and Google Scholar databases were used to identify relevant peer-reviewed research articles describing evaluations of hemorrhage control courses for LFR between 2013 and 2024. Studies examined whether the training course was implemented in low- and middle-income countries (LMICS); trainees were LFR and had outcome measures, including knowledge, skills, attitudes (confidence gained, willingness, comfort, and likelihood to intervene) toward care utilization for trauma patients, as well as outcomes.

**Results:**

The SR included 12 articles. The quality of the selected studies was generally high. Five studies (42%) reported improved willingness, confidence, comfort, and the likelihood of responding safely to trauma. Nine studies (75%) used pre-and post-tests to document knowledge acquired, and five studies (42%) used knowledge retention evaluation at different intervals and reported substantial knowledge and skill reductions after a few months (1–3 months, 3–6 months, or both 6 and 9 months or up to 3 years). A few studies followed incident reports to document the utilization of knowledge and skills acquired during training. None reported patient outcomes.

**Conclusion:**

Bleeding control training courses for layperson first responders in LMICS significantly improve knowledge, skill, confidence, and willingness to intervene to apply bleeding control techniques to trauma patients. Evaluating clinically relevant outcomes is needed to strengthen the evidence.

**Supplementary Information:**

The online version contains supplementary material available at 10.1007/s00068-025-02917-4.

## Introduction

Exsanguinating bleeding is the most common cause of preventable trauma death due to the inability to control bleeding at the injury scene, and it often happens within the first two hours of the injury [1]. Moreover, over 50% of hemorrhage-related deaths occur in the prehospital setting, with 93% of them happening in LMICs, reflecting the increasing burden of trauma in these countries [2]. Therefore, it is vital to empower laypeople first responders, such as commercial drivers, police officers, relatives, and bystanders, to respond to injured patients'survival needs, particularly bleeding control [3–5]. Implementing a Lay First Responder (LFR) program in low- and middle-income countries (LMICs) is the best starting point to address this issue.

In North America, these courses have become more widespread as the'STOP THE BLEED'[STB] course, since October 2015, and our institution has already conducted a systematic review to analyze the effect of the STB training course on the knowledge, skill, and attitudes of lay first-person responders for hemorrhage control [6]. Callese et al. [7] conducted a similar review of trauma educational initiatives for layperson first responders in resource-poor settings. A scoping review on prehospital hemorrhage management has been published recently, but it was not limited to managing traumatic exsanguination [8]. A systematic review of hemorrhage control courses in LMICs has not yet been conducted in trauma settings.

We hypothesized that hemorrhage control training courses improve LFR Knowledge, skills, and attitudes toward hemorrhage control in LMICs. This systematic review analyzed the effect of hemorrhage control training courses for LFR on their knowledge, skill acquisition, and attitude (willingness, confidence, comfort, and likelihood to intervene) and control bleeding in trauma patients.

## Methods

### Information sources and searches

We conducted a literature search using PubMed/MEDLINE and Google Scholar to identify relevant peer-reviewed studies published between December 1, 2013, and May 31, 2024. We used the Preferred Reporting Items for Systematic Reviews and Meta-Analyses (PRISMA) guidelines (Supplementary file 1). For the PubMed/MEDLINE search, we used the following MeSH and free text terms:"Wounds and Injuries"[MeSH]; Trauma; Emergency;"Education"[MeSH];"Training Programs"[MeSH]; Course; Training;"Community Health Workers"[MeSH];"Volunteers"[MeSH];"First Aid"[MeSH];"First Responders"[MeSH]; Community; Layperson; Lay First Responder;"Hemorrhage"[MeSH];"Blood Loss"[MeSH]; Bleeding Control; Developing Countries [MeSH]; and Low and Middle-Income Country. We used the filters for text availability (full text); article type (clinical study, clinical trial, comparative study, evaluation study, observational study, and randomized controlled trial); publication date (1 December 2013 to 31 May 2024); species (humans), and article language (English). The search yielded 4,187 articles. We also performed a comprehensive search on Google Scholar using keywords such as"Trauma Care,""Emergency Response,""Training Programs,"“Education Programs,"“Layperson,"“Community First Responders,"“Bleeding Control,"“Hemorrhage Control,"“Low and Middle-Income Country,"and “Low Resource Setting."The search was limited to articles published between 2013 and 2024, which generated 19 articles (Supplementary file 2). Afterward, we reviewed the titles of these articles to evaluate their relevance. After removing duplicates, the relevant titles recognized from the Google Scholar search were merged into the PubMed search results for further evaluation. This approach warranted a thorough assessment of pertinent literature for inclusion in the systematic review. To ensure comprehensive coverage, we conducted a bibliographic search that examined the reference lists of relevant articles.

#### Study selection

After removing duplicates and irrelevant studies, two independent reviewers screened the titles and abstracts for inclusion in the full-text screening phase. They then conducted in-depth evaluations of each full-text article to determine its inclusion in the systematic review. In disagreement, a third reviewer independently assessed all titles, abstracts, and full-text articles and made the final decision.

#### Inclusion/exclusion criteria

Studies included in the systematic review were original studies, such as observational studies, follow-up surveys, and randomized controlled trials, that focused on trauma education, training, or courses provided to laypersons. Laypersons were defined as community members, school employees, students from schools or colleges, medical and nursing students, law enforcement officers, security professionals, and commercial vehicle drivers. In contrast, healthcare professionals were excluded as they do not represent laypersons, which is the focus of this survey. These studies were published in English between January 1, 2013, and May 31, 2024, and were available as full texts. We excluded the American College of Surgeons ‘s STB courses because they are reliant on the availability of relatively expensive commercial tourniquet tools; however, the current review focused on hemorrhage control courses that used indigenous materials that are low-cost and locally available in the setup of LMICs to ensure the sustainability [9, 10]. Previous work has shown that most STB courses are conducted in high-income countries [6].

Non-original articles, such as books, documents, reviews, commentaries, letters, news articles, personal narratives, patient education handouts, case reports, and legislation, were excluded. In addition, retracted publications, studies reporting only the epidemiology of trauma patients, psychological studies, and pilot studies were excluded. Non-English articles and articles without full texts were also excluded.

The World Bank country classification depends on estimated Gross National Income per capita data in U.S. dollars (converted from local currencies). Countries are categorized as low, middle (further divided into upper and lower), and high-income countries [11].

#### Definitions

 Low- and middle-income countries, defined by the World Bank as low- and middle-income countries (LMICs), are nations with a gross national income (GNI) per capita between $1,136 and $4,465 (as of 2024). These countries fall between low-income and upper-middle-income economies in terms of economic development and income levels [11].

Willingness is measured by self-reported yes or no responses to direct questions in pre- and post-training questionnaires [12].

Confidence/Comfort/likelihood to intervene are defined based on self-reported measurements on a Likert scale in response to a pre/post-training questionnaire [12], both willingness and self-reported confidence/comfort/Likelihood to intervene reflect the attitude of laypersons toward hemorrhage control [12].

#### Outcomes

- Acquired knowledge, skills, and the retention of knowledge (assessed through test scores)
- Affective outcomes include willingness, confidence, comfort, and/or willingness to intervene - perform trauma first aid, including hemorrhage control.

#### Data extraction and synthesis

The data extracted from the studies include the study objective, design, population, intervention and control details, outcomes measured, and key findings. We synthesized the findings from the included studies in a summative and tabular manner. The tabulation included details such as the year of publication, location of the study, study population, sample size, study objectives, study design, and measured outcomes, including knowledge, skill, willingness to perform bleeding control, confidence, comfort, likelihood to intervene, retention of knowledge or skill, and patient-related outcomes.

#### Assessment of study quality

The Newcastle–Ottawa Scale (NOS) was used to evaluate the methodological quality and risk of bias in the included studies, focusing on three key aspects: selection of study groups, comparability of groups, and assessment of outcomes or exposures. Two reviewers independently evaluated each study, and disagreements were resolved through discussion or consultation with a third reviewer.

## Results

**Fig. 1:**
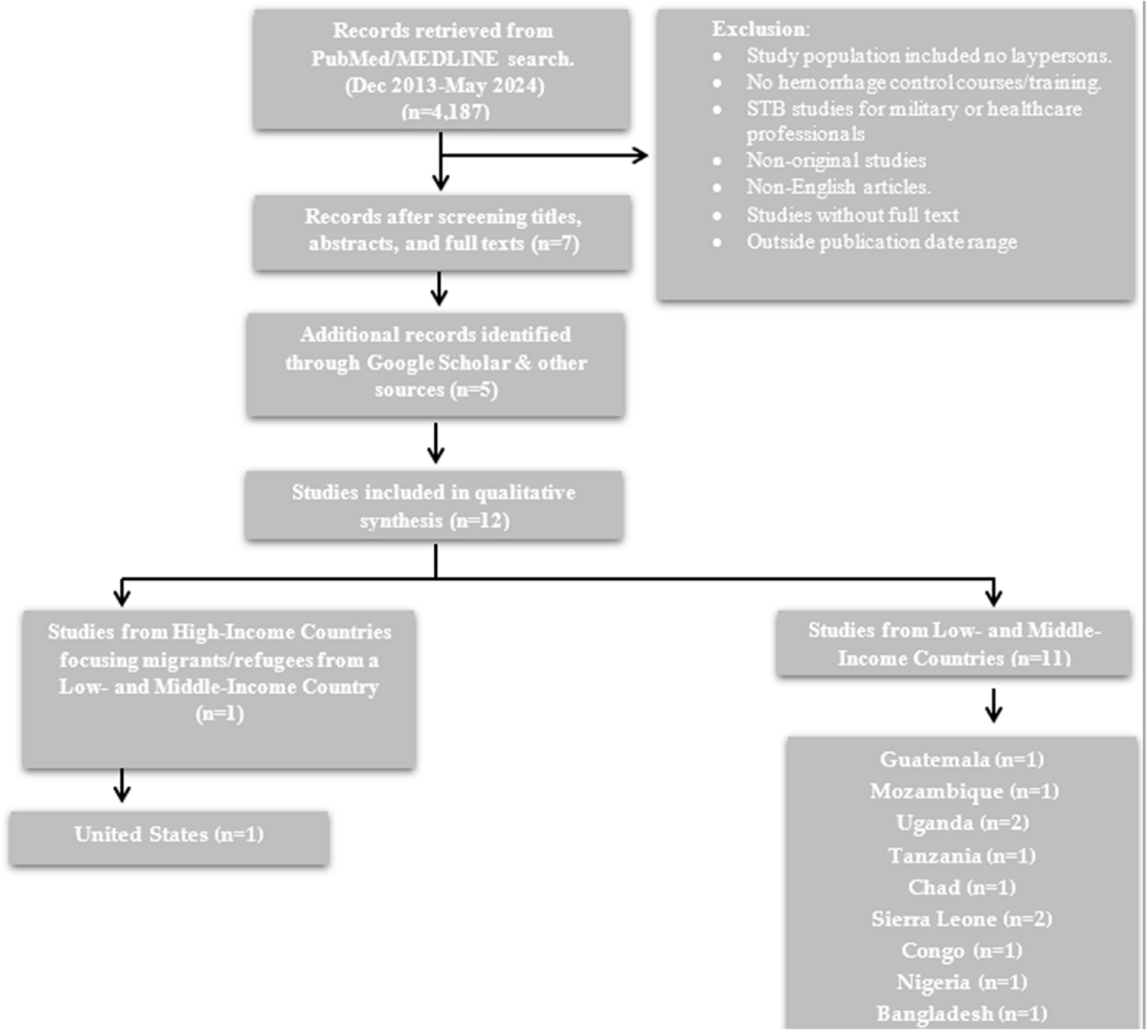
Studies selection process for systematic review

After a thorough evaluation, seven papers out of 2.298 were deemed eligible from the PubMed/MEDLINE database search and included in the final analysis. Subsequently, an exhaustive search on Google Scholar yielded four articles, and the bibliographic search identified one additional relevant paper. The systematic review included 12 articles that met the inclusion criteria for the final analysis [13–24] (Fig. 1).

### Study Characteristics

Tables 1 summarize the 12 studies that met the inclusion criteria [13–24]. Out of these 12 articles, nine originated from the African continent [13, 15–20, 22, 23], with two from Lower- Middle-Income Countries, Nigeria [23] and Tanzania [22], and seven from low-income Sub-Saharan Africa, namely Mozambique [20], Uganda [13, 15], Chad [19], Sierra Leone [16, 18], and Congo [17]. One study was published from an Upper-Middle-Income country in Latin America, Guatemala [14]. In another study published in Bangladesh [21], a Low-Middle-Income Country in Asia, volunteers responded to 100% of calls for more than 1,500 crashes over 6 years, indicating high levels of comfort, willingness to intervene, and confidence in delivering necessary care, including hemorrhage control. Still, the paper documents the success of their initiative in using a mobile application (Traumalink) and a volunteer network of laypersons to attend calls through a dedicated full-time call center and provide first aid care in the retrospective study. Still, the attitude part was not clearly stated. Most African studies mainly targeted drivers of commercial passenger vehicles, including motorcycle taxi drivers [13, 15, 19, 23]. One study from the USA was included, as it focused on laypersons from an African (Somali) minority community [24].

**Table 1:** Summary of lay first responder training programs in low- and middle-income countries: characteristics, outcomes, and impact

Country	Author (Year)	Target population (*N*)	Training duration (Survey Qs)	Key skills taught	Knowledge/skill improvement	Attitude (Confidence/Willingness and Confidence to Intervene)	Retention/follow-up	Patient outcome/evidence of utilization
Nigeria	[23]	Commercial drivers (128) (62 intervention + 66 control) [pre-posttests; comparative study]	2 days (24 Qs)	General knowledge of first aid, safety at a crash scene, initial assessment of the crash victim, rapid survey/focused examination of the crash victim,	Intervention group: Knowledge: 49% → 58%; Skills: 18% → 81%	Not reported	3 months Knowledge: 49% (baseline) → 59% (3 months); Skills: 18% → 72% (3 months)	No direct clinical outcomes were reported. Significant improvement in first aid knowledge and skills of intervention drivers. A slight drop in skills scores occurred 3-month post-intervention
Mozambique	[20]	Hospital staff (43) & laypersons (45) [pre-posttests; comparative study]	2.5 h (12 Qs)	ABCD, scene management, transport, splinting	Laypersons: 27% → 50%; Hospital staff: 42% → 60%	Not reported	Not reported	No direct clinical outcomes were reported. Increased the number of capable responders
Uganda	[13]	Motorcycle taxi drivers (121/154) [pre-posttests; no control group]	5 h (15 Qs)	Bleeding control, scene management, airway and breathing, recovery position, and victim transport	Bleeding control: 57% → 80%; Scene management 38% → 60%; Airway and breathing 43% → 52%; Recovery position 13% → 43%; Victim transport 88% → 94%	70/76 (92%) confident after using skills	9 months follow up (110/154) but no retention study; 76 out of 110 (69%) used at least 1 of the 5 first aid skills taught	No clinical outcomes were reported. First aid used: airway 11.5%, bleeding/bandaging 55.2%, splinting/wrapping 13.8%, movement/positioning 23.0%
Guatemala	[14]	Civilians, law enforcement, firefighters (287) [pre-posttests; no control group]	5 h (26 Qs)	Scene safety, Airway, Bleeding (specifically Hemorrhage Control), and Triage	Mean test score: 43% → 68%; p < 0.001)	Not reported	No follow-up yet	No clinical outcomes were reported. The authors plan to conduct a 12-month follow-up to measure knowledge retention and skill decay
Tanzania	[22]	Leaders from traffic police and drivers'associations (12) [Qualitative study]	Not applicable; Explored views on potential training processes, methods, and materials	Not applicable; the study assessed views on training only. Stakeholders emphasized the need for practical skills and basic knowledge of pre-hospital emergency care	Not measured. A qualitative study exploring stakeholder perspectives	Stakeholder perspectives reported. Stakeholders viewed training as likely to boost the confidence of potential trainees. They discussed factors influencing motivation/willingness (perceived benefits, incentives)	Not applicable; Stakeholders recommended frequent refresher training to maintain knowledge and skills	Not applicable; qualitative study from stakeholder perspectives. Training is seen as improving survival outcomes and public respect
Chad	[19]	136 LFRs: 108 drivers, 15 NGO, 13 Red Cross. Pre/post-test on 106 drivers. Follow-up: 49 trained, 51 untrained drivers. [Pre-posttests; Comparative study]	5 h (15 Qs)	Scene safety, bleeding, splinting, airway	Significant knowledge gain (P <.001) in Scene Safety (33% → 59%), Airway (25% → 44%), Bleeding Control (35% → 53%). Minimal change in Transport (81% → 83%).)	**Confidence:** Trained LFRs reported avg. 8.5/10 (SD = 3.01, *n* = 38); 98% still confident at 12 mo. **Willingness**: 100% of untrained drivers are interested in training; 96% of untrained willing to pay for training. **Motivators:** helping others, new skills, status, and customer acquisition	12-month follow-up via surveys/interviews on motivations, skill use, and confidence. No 12-month re-testing for knowledge retention. Stakeholders advised frequent refresher training	No clinical outcomes were reported. Over the course of 6 months, LFRs treated 71 patients in 38 incidents. Skills used: airway 32%, bleeding/bandaging 61%, splinting 45%, transport 82%. At 12 months, 97.9% reported using skills
Sierra Leone	[18]	LFRs (4,529 trained; 465 tested) [Pre-posttests; No control group]	5 h (23 Qs)	First aid principles include scene management, airway and breathing management, hemorrhage control, fracture splinting, and victim transport. No CPR	Significant knowledge gain: median score improved from 34.8% to 78.3% (p < 0.05). All categories improved: Scene (43.4% → 74.2%), Airway (37.4% → 78.1%), Bleeding (21.1% → 77.1%), Splinting (39.1% → 69.5%), and Transport (29.8% → 75.7%)	Not reported	Knowledge decay was assessed at 6 and 9 months. Median scores: 60.9% at 6 months, 43.5% at 9 months—both significantly higher than the pre-test (34.8%), showing retained knowledge despite some decay	No clinical outcomes were reported. Over six months, LFRs treated 1,850 patients. Skills used: airway/breathing 20%, hemorrhage/bandaging 51.3%, splinting 33.9%, transport 88.3%. Motorcycle injuries are most common (48.7%)
Uganda	[15]	Motorcycle taxi drivers (Original training in July 2016: 154 drivers trained. Follow-up in July 2019: 115 trained & 124 untrained drivers [Mixed methods; Comparative study]	5 h (Follow-up used 7-Qs for trained,3-Qs for untrained	Bleeding control, scene management, airway and breathing management, recovery position, and transport. Skills included spinal immobilization, wound dressing, splinting, and ABCs	This study was a follow-up focusing on the perceived benefits and continued involvement of trained LFRs 3 years later	**Confidence:** 98.3% of trained LFRs still felt confident in using their skills three years post-training Key motivator. Interest: 95.2% of untrained drivers wanted training; 64.5% were willing to pay for training	Mixed-methods follow-up in July 2019, 3 years post-training. Used surveys and interviews. A prior study [13] included a 9-month knowledge retention assessment	No clinical outcomes were reported; 96.5% of trained LFRs reported using skills. Helping others motivated continued involvement. Trained LFRs noted an identity shift, increased respect, and a 24.4% higher median income compared to untrained individuals
Sierra Leone	[16]	Evaluated LFR program surveying 90 hospital providers (50 Makeni (intervention location), 40 Kenema, control location) to assess prehospital care quality. [Pre-posttests; Comparative study]	5 h (7 Qs) (PETCAT)	Scene and airway management, bleeding control, splinting/immobilization, transport	PETCAT scores: Makeni rose from 0.51 to 5.15/10 in 14 mo (*p* < 0.0001); Kenema minimal change (1.0 to 1.24)	There is no direct assessment of LFR or provider attitudes. PETCAT measures providers'perception of intervention frequency and quality	A longitudinal evaluation comparing provider PETCAT assessments from 1 month pre-launch to 14 months post-launch. There is no data on individual LFR retention over time	No clinical outcomes were reported; however, the rise in PETCAT score (from 0.51 to 5.15) indicates an increase in prehospital intervention frequency and quality among hospital providers
Bangladesh	[21]	Community volunteers (599 trained) [a retrospective, descriptive, longitudinal analysis of an operational program]	Full day	Trauma first aid program (specific skills taught to the volunteers are not detailed)	Evaluated program’s operational reliability and efficiency as proxies for clinical outcomes; lacked detailed patient clinical data. No reported volunteer knowledge or skill assessment results	No information was provided on the attitudes, confidence, or willingness of volunteers or community members	Volunteer retention was assessed over 6 years (November 2014–November 2020); however, the retention findings were not detailed in the study	No clinical outcomes were reported. Evaluated operational metrics as proxies for clinical outcomes due to lack of patient data: response rate, crash-to-VFR arrival time, transport rate to hospital, crash-to-hospital arrival time, and alignment of injury severity with patient disposition. Benefits of prehospital trauma care acknowledged
Congo	[17]	Community health workers (42) [mixed methods; No control group]	3 days	Pilot WHO CFAR course assessed: 7 lectures, case discussions, 15 skills stations; no CPR included. Focused on basic first aid for community health workers	FGDs highlighted that hands-on, practical skills training was key to participant satisfaction and perceived course quality. Instructors reported 90–95% of learning objectives were met, with participants gaining lifesaving skills	Self-confidence in first aid skills increased significantly from 17.9% before training to 95.3% after training (*p* < 0.001)	This pilot study evaluated the initial training only; no information was reported on volunteer retention or follow-up beyond the 3-day training period	No clinical outcomes were reported This small-scale feasibility study evaluated the pilot course's perceived outcomes—knowledge and confidence gain—without reporting actual skill use in the field or patient outcomes

### Study objectives

In the final analysis, the studies covered various objectives related to first-aid training and trauma management. Among the objectives, some aimed to assess the impact of first aid training on the knowledge and skills of commercial drivers [19, 23] to evaluate the feasibility of implementing modified trauma resuscitation training for hospital personnel and laypersons [16, 20], to assess the knowledge, self-efficacy, and trust among participants and first responders after a culturally adapted bleeding control program [21], and to create a sustainable prehospital lay first responder programs [13]. Moreover, some studies evaluated post-crash first aid (PFA) educational programs for police officers [22], the efficacy of LFR programs using validated pre-post-tests [19], the long-term social and financial effects of training programs on participants [15], and the frequency and quality of LFR prehospital intervention [16]. The Asian study investigated the feasibility and impact of a community-based trauma first-aid program using volunteers [21].

### Evaluation of the programs

In 9 studies, the pre-and post-test was the most used evaluation tool [13, 14, 16, 18–23]. Five studies included a retention evaluation at specific follow-up intervals: 3 months (72.3% retention) [23], 6 months (60.9% retention) [18], 9 months (43.5% retention) [13, 18], and 3 years post-training [15], where 96.5% of participants reported applying their skills with confidence and noted social and financial benefits [13]. One study assessing post-crash first aid training showed a significant increase in mean knowledge scores from 44.73% to 72.92% at 6 months [22]. Another study evaluating law enforcement personnel showed improvements in post-training hemorrhage control from 31.8% to 67.9%, with better performance among civilians than firefighters (*p* < 0.001) [14] (Table 1).

Five studies evaluated different aspects of the WCCL spectrum, including willingness, confidence, comfort, and likelihood of intervening or responding to a bleeding patient. For example, a follow-up study of motorcycle taxi drivers in Uganda found that 110 of 154 trainees were retained at 9 months, with 70 of 76 feeling'confident'in using at least one first-aid skill [13]. Similarly, a Sierra Leone study showed that 61.2% of participants reported applying bleeding control techniques in real-life scenarios [18] (Table 1).

### Quality assessment

Six studies demonstrated high methodological quality with robust scores (7–9). Four studies scored 8 out of 9, indicating strong adherence to methodological criteria. Additionally, two studies scored 7 out of 9, signifying a high level of methodological quality with a low risk of bias (Table 2). Overall, the quality assessment of the included studies indicates a generally high level of methodological rigor, with most studies demonstrating robust selection, comparability, and outcome assessment methods.

**Table 2:** Methodological quality and risk of bias for studies based on the Newcastle–Ottawa Scale (NOS)

Study	Selection	Comparability	Outcome	Score
[23]	3	2	3	8/9
[20]	3	2	1	6/9
[13]	3	2	2	7/9
[13]	4	2	2	8/9
[14]	3	1	2	6/9
[22]	2	1	1	4/9
[19]	4	2	2	8/9
[18]	3	2	2	7/9
[16]	4	2	2	8/9
[24]	4	2	2	8/9
[21]	3	0	2	5/9
[17]	4	0	2	6/9

## Discussion

This systematic review found that first-aid bleeding control training courses for layperson first responders increase the knowledge, skills, self-reported confidence, and willingness to participate (positive attitude effect) in immediate bleeding control for trauma patients in many countries; the majority of included papers belong to the LMICs categories were stop the bleed is taught as part of modified trauma first aid curriculum developed in Guatemala and endorsed by the WHO [1]. One report from the USA (HIC) focused on minority groups with difficulty in communication and access [24], while in the rest of the reports, the focus was on the tourniquet application training outside the classic American College of Surgeons- STB course [25–29] were excluded. We considered synthesizing the positive attributes, such as willingness and confidence, across the included studies. However, that was hard as the heterogeneity in reporting and extracting such details was evident, and there was no standard way to report such aspects of training outcomes,for example, while other studies used yes/no self-reported responses to a questionnaire to document the willingness as compared in the pre and post-training questionnaires, self-reported confidence/comfort and willingness to intervene when faced with traumatic bleeding situations is scored based on 5-Likert scale. The reported papers in this review did not define these attributes. However, these attributes or indicators were so distinct that we could not find a tool to combine them into a single analysis or metric.

However, data on the impact of such training of layperson first responders on patient-related clinical outcomes and the experience of layperson training in other regions of the globe, principally Asia and Europe, are underreported in the included decade.

Furthermore, some studies have observed and reported knowledge and skill decay through follow-up surveys. Locally available first aid materials were used, and follow-up on actual implementation, as indicated by the incidence reports, is helpful for further evaluation. There is a need to refresh knowledge through follow-up courses, develop a critical mass of trainees through the"train the trainer"model, stock used materials, and target certain layperson groups, for example, taxi drivers as an excellent primary layperson target with reported financial and social gains to the providers and disciplined laypersons like police and civil defense officers in LMICs as well as high-income countries (HICs). Furthermore, there is an urgent need to establish local registries and develop networks to further advance the program in establishing a trauma system in these underserved countries. In place of this demand, a new study from a sub-Saharan LIC, Cameroon, demonstrated that bystander interventions reported as received prehospital care group are associated with decreased emergency department mortality in injury victims, so we agree with the author's conclusion that implementing a LFR training will improve frequency and quality of care and potentially improves survival demonstrate that further capacitating, them through a LFR hemorrhagic training course, will further reduce this mortality [30].

These findings align with prior observations and recommendations on how to improve trauma care, as outlined in the WHO Guidelines for Essential Trauma Care and the Disease Control Priorities Project [10, 31].

Concerning attitudes toward the provision of bleeding control interventions, most papers documented the positive impact of interval surveys. Surveys reported confidence scores, self-efficacy with a positive impact related to previous training in one paper, and social/financial gains that motivate involvement in training and provision of bleeding control; an important area that needs exploration to ensure the sustainability of these efforts as part of the global need to prioritize care [32]. Similarly, a recent paper from the USA explored the layperson first care provider in simulated mass causality demonstrated a positive willingness to lead interventions and effective intervention for trained groups and plausible explain the effect on two reasons,the first is that training provides a framework for responding (sense of control) and the second training lessens uncertainty thus enables decision making [33].

Furthermore, this review urges the involvement of local stakeholders to identify local needs from a trauma perspective and cultural aspects and modify available programs for “stop the bleed” first aid.

No study could explore the impact on outcomes (Table 1), although some could report utilization using incidence reports and quality indicators. The evidence of utilization of what LFR learned can be regarded as a surrogate for outcome reporting, although it is not a direct patient-related outcome. Nevertheless, it potentially increases the odds of better outcomes by ensuring immediate skill application to maintain life and assure safer and quicker transportation to healthcare facilities. Orkin et al. [34] conducted a recent systematic review on layperson first aid training for emergency care in underserved populations and low-resource settings, including trauma and non-trauma emergencies, concluding the possible impact on outcomes and community emergency management capacity. This review included a few trauma papers, but it does not comment specifically on the attitude of provision. Other reports published in a period outside the timeframe of the present review demonstrated mortality benefits for prehospital BLS care,in landmine prevalent areas of Iraq and Cambodia (published in 2003), a five-year prospective study for a model of prehospital care paramedics training rural lay first responders with reported mortality decrease from 40% to 14.9% [35]. The same group demonstrated in 2012 a similar impact of the model of LFR for severe road traffic injuries-related mortality (the treatment group is 8% compared to 44% in the control group) [36]. Nevertheless, those papers reported on several different BLS and prehospital interventions that did not focus on hemorrhage control, and LFRs were not the main or solo participants.

Training LFRs is cost-effective, as highlighted by Delaney et al. [37], and investment in basic or intermediate prehospital care can be comparable in cost-effectiveness to more widely implemented health interventions. However, the current review does not evaluate the cost-effectiveness of LFR in low-income countries, which could be a determinant factor in low-source countries.

### Strengths and weaknesses of the review

After evaluating these programs in various contexts, it was observed that first-aid training effectively enhances knowledge, skills, and self-assurance. However, maintaining the acquired skills and exploring the WCCL spectrum pose challenges that emphasize the necessity for continuous research and educational program development in trauma in LMICs.

We applied a standard systematic review methodology to assess three central outcomes from hemorrhage control training (as a part of modified trauma first aid): the acquisition & retention of knowledge and skill, attitude toward providing first aid bleeding control (Willingness, Confidence, Comfort and Likelihood to intervene) with reported influencing factors and patient outcomes. Though this study built on earlier work by Callese et al. [7], it is different as it focuses solely on training that only included the recently implemented modified trauma courses and was limited to'trauma-naïve'layperson first responder populations in LMICs and one study from HIC that reported a minority group from African origin as well as exploring the attitude effect [24].

We acknowledge a potential publication bias, as we only included English studies, and that there needs to be reported experiences from a broader range of countries. The fact that we did not include non-English studies could be one reason why Europe and Asia were underrepresented in the current review. Furthermore, there is apparent heterogeneity among the included papers due to the different setups, trainers'backgrounds, trainees, use of varying time frames and evaluation tools, and timing of training and follow-up checks on retention of gained knowledge, skills, and attitudes. Nevertheless, the feasibility of short courses and their effect on at least two of the examined domains were consistent in LMICs and HICs focusing on minority communities. For example, in the USA, where around 8% of the population do not speak fluent English, restraining their access to potentially lifesaving knowledge and skills as well as communication with immediate responders corresponding to the civil defense, police, and Emergency Medical Services (EMS) during prehospital emergencies [24].

From a task-shifting or force multiplier standpoint, this review supports more'train the trainer'possibilities and options that utilize lay, non-medical faculty to further spread valuable bleeding control knowledge and skills within various communities. The documented decay in knowledge and abilities calls for regular retraining, as reported by the Bangladeshi paper (half-day retraining every 6–12 months), which is likely every 6 months based on the current evidence. Geographically, this review reveals marked regional gaps in the locations of published evaluations of HC courses. For this review, no studies were included from the European Region (EUR), and only one study was included from the Southeast Asian Region (SEAR).

Any bleeding control course must demonstrate that it reduces the death toll from preventable deaths due to uncontrolled traumatic exsanguination. In the meantime, while we await definitive data showing the survival benefit of bleeding control courses, the best evidence from this review should suffice to inform the more targeted and focused implementation of bleeding control courses for laypersons. We aimed to guide program developers and policymakers toward interventions that are subject to evaluations with demonstrable health effects and to help researchers and evaluators understand and optimize these initiatives. There is a need to standardize attitude measurement and define related aspects, such as willingness, confidence, comfort, and likelihood to intervene. The heterogeneity in reporting calls for a consensus on how to report and measure this domain; a Likert scale seems preferable to a yes/no approach, as it allows for a deeper understanding and documentation of these human aspects, which are otherwise difficult to quantify and measure. While methodological differences—such as variations in questionnaire design, scoring systems, and outcome reporting—introduced substantial heterogeneity, the lack of standard deviation reporting in four out of six studies further limited the ability to conduct a meaningful meta-analysis. Additionally, only a few studies included control groups, and these varied considerably in composition—from hospital staff to laypersons, which also restricted the ability to pool intervention versus control group differences.

## Conclusion

Courses for layperson first responders in LMICS would significantly improve knowledge, skill, confidence, and willingness to intervene to apply bleeding control techniques to trauma patients. There is a need to retrain trainees at intervals of 6 months to maintain and reinforce their knowledge and skills. The evaluation of clinically relevant patient outcomes, precisely their effect on preventable deaths from trauma and bleeding, is needed to strengthen further the evidence behind the recommendations for the more widespread teaching of laypersons.

## Supplementary Information

Below is the link to the electronic supplementary material.

Supplementary file1 (DOCX 33 KB)

Supplementary file2 (DOCX 15 KB)

## Data Availability

No datasets were generated or analysed during the current study.
