# Supplementary material for: The effect of external bleeding control training courses on lay first-person responders'knowledge, skills, and attitudes in low- and middle-income countries: a systematic review
Source: Eur J Trauma Emerg Surg. 2025 Jul 14;51(1):252. doi: 10.1007/s00068-025-02917-4 (PMC12259789; doi:10.1007/s00068-025-02917-4)
Supplement: Supplementary file 2 — Supplementary file2 (DOCX 15 KB) [file 68_2025_2917_MOESM2_ESM.docx]

| Search Strategy and Article Retrieval from PubMed/MEDLINE & Google Scholar (2013–2024) | | | |
| --- | --- | --- | --- |
| Database | Search Query | Filters Applied | Total Articles Retrieved |
| PubMed/MEDLINE | Trauma [Title/Abstract] AND "Training Programs" [Title/Abstract] AND "Layperson" [Title/Abstract] AND ("Bleeding Control" [Title/Abstract] AND ("Low and Middle-Income Country" [Title/Abstract] OR "Low Resource Settings" [Title/Abstract])) | Filters: MEDLINE, Date range: 2013/12/01 - 2024/05/31, Sorted by: Publication Date | 4187 |
| Google Scholar | ("Trauma Care" OR "Emergency Response") AND ("Training Programs" OR "Education Programs") AND ("Layperson" OR "Community First Responders") AND ("Bleeding Control" OR "Hemorrhage Control") AND ("Low and Middle-Income Country" OR "Low Resource Settings") | Date range: 2013–2024 | 19 |
